# Area-Level Sociodemographic Differences Between Indian Health Service Purchased/Referred and Non-Purchased/Referred Care Delivery Areas

**DOI:** 10.3390/ijerph23050622

**Published:** 2026-05-08

**Authors:** Sarah H. Nash, Rachael Adcock, Chi Wang, Mindy C. Hebert-DeRouen, Natalie S. Joe, Dornell Pete, Tyler B. Kratzer, Charles L. Wiggins, Lihua Liu, Bradley D. McDowell

**Affiliations:** 1 Holden Comprehensive Cancer Center, University of Iowa, Iowa City, IA 52242, USA; bradley-mcdowell@uiowa.edu; 2 Department of Epidemiology, College of Public Health, University of Iowa, Iowa City, IA 52242, USA; 3 University of New Mexico Comprehensive Cancer Center, Albuquerque, NM 87131, USA; raadcock@salud.unm.edu (R.A.);; 4 Department of Internal Medicine, School of Medicine, University of New Mexico, Albuquerque, NM 87131, USA; 5 Department of Public Health Sciences, New Mexico State University, Las Cruces, NM 88003, USA; 6 Department of Epidemiology and Biostatistics, University of California San Francisco, San Francisco, CA 94143, USA; 7 Division of Cancer Control and Population Sciences, National Cancer Institute, Bethesda, MD 20850, USA; 8 Cancer Prevention Fellowship Program, Division of Cancer Prevention, National Cancer Institute, Bethesda, MD 20850, USA; 9 Epidemiology Program, Public Health Sciences Division, Fred Hutchinson Cancer Center, Seattle, WA 98109, USA; 10 Surveillance & Health Equity Science, American Cancer Society, Atlanta, GA 30303, USA; 11 Los Angeles Cancer Surveillance Program, University of Southern California, Los Angeles, CA 90089, USA; lihualiu@usc.edu

**Keywords:** Native American, American Indian and Alaska Native, Indian Health Service, descriptive epidemiology

## Abstract

**Highlights:**

**Public health relevance—How does this work relate to a public health issue?**

- Cancer statistics for American Indian and Alaska Native (AIAN) people are often restricted to Purchased/Referred Care Delivery Area (PRCDA) counties, to address racial misclassification.
- This study explores county-level differences in socio-economic, demographic, and health-related factors between PRCDA and non-PRCDA counties, in order to describe contextual differences for those that are and are not included in AIAN cancer statistics.

**Public health significance—Why is this work of significance to public health?**

- Understanding differences in cancer burden between AIAN peoples and their non-Native counterparts is critical to the pursuit of health equity.

**Public health implications—What are the key implications or messages for practitioners, policy makers and/or researchers in public health?**

- The data presented herein indicate variability in county-level sociodemographic characteristics, area-based social measures, and prevalence of health outcomes between PRCDA and non-PRCDA counties.
- While these data do not speak directly to the AIAN experience, they provide critical contextual information to understand whether the exclusion of non-PRCDA AIAN people from cancer statistics may bias estimates.

**Abstract:**

Purpose: Purchased/Referred Care Delivery Area (PRCDA) counties are those where resident American Indian and Alaska Native (AIAN) people are eligible for Indian Health Service care. Due to concerns about racial misclassification, cancer statistics for AIAN people are often restricted to PRCDA counties. Differences in sociodemographic characteristics may exist between PRCDA and non-PRCDA counties, but have not been described; therefore, the potential selection bias associated with the restriction to PRCDA counties remains unknown. Methods: We used data from the University of California, San Francisco Health Atlas to explore ecological differences in county-level demographic, socioeconomic, healthcare access, and health outcomes data between PRCDA and non-PRCDA counties (n = 3152 counties). We tested for statistical differences in mean levels of demographics between PRCDA and non-PRCDA counties using Pooled or Welch *t*-tests. Results: We observed small, but statistically significant differences between PRCDA and non-PRCDA counties in county-level demographic and socioeconomic characteristics (age, poverty, utility services threat, unemployment, educational attainment, computer access, and median income), neighborhood and environment characteristics (overcrowding, severe mortgage/rent burden), healthcare access and utilization (uninsured, annual checkup, annual dental visit, mammography, binge drinking, smoking, physical inactivity, social isolation), and health outcomes (poor mental health, arthritis, poor self-rated health, high blood pressure, diabetes, high cholesterol, and obesity). Conclusions: These results indicate variability in county-level measures between PRCDA and non-PRCDA counties. While these data do not speak specifically to AIAN peoples’ experiences, they provide critical contextual information to understand how exclusion of AIAN people residing in non-PRCDA counties from cancer statistics may bias risk estimates.

## 1. Introduction

There is substantial heterogeneity in cancer incidence, mortality, and survival among AIAN peoples [1,2]. Differences are most commonly investigated by the Indian Health Service (IHS) region, because these six regions—Alaska, Northern Plains, Southern Plains, Pacific Coast, Southwest, and East—not only reflect how health services for AIAN individuals are managed across the U.S., but also broadly represent regional differences in modern AIAN cultures [1,2,3]. However, there are also differences in cancer burden between Purchased/Referred Care Delivery Area (PRCDA, i.e., those counties directly served by IHS, or IHS-adjacent) and non-PRCDA counties [4,5].

Differences in cancer burden between PRCDA and non-PRCDA counties have been less thoroughly investigated because of concerns for under-identification of AIAN peoples in non-PRCDA counties [6,7]. While AIAN identification is markedly improved in cancer registries through linkage with IHS records, this linkage is less effective in non-PRCDA counties than it is in PRCDA counties [4]. Given that a more inclusive approach to reduce AIAN misclassification has not been available, studies have sidestepped the issue by presenting data only for AIAN people residing in PRCDA counties [2,8]; resulting in systematic omission of AIAN people residing in non-PRCDA counties. Therefore, very little is known about cancer and associated risk factors among AIAN people resident in non-PRCDA counties; which is critical, given that the 651 PRCDA counties represent only 20.7% of the 3141 counties in the United States, and only 53.3% of all AI/AN persons resided in those PRCDA-designated counties from 2013 to 2017 [9] leaving a large proportion of AIAN people unrepresented in national statistics.

Despite data quality concerns, it is important to understand the likely stark differences in cancer burden and treatment between PRCDA and non-PRCDA counties. However, because differences in sociodemographic characteristics of PRCDA versus non-PRCDA counties have not been comprehensively described, the potential selection bias associated with restricting studies and/or national statistics to AIAN people residing in PRCDA counties remains unknown. In this study, we conducted ecological analyses of county-level social and environmental measures from the University of California, San Francisco (UCSF) Health Atlas between PRCDA and non-PRCDA counties. While these data do not directly speak to the experience of AIAN people, they describe contextual differences in residence between those living in PRCDA and non-PRCDA counties.

## 2. Materials and Methods

### 2.1. Data Source and Data Availability

This study used an observational ecological design at the county level. County-level data were downloaded from the UCSF Health Atlas, a dashboard and repository of data on area-level characteristics, as of 2024, including data for all 50 states, the District of Columbia (DC), and Puerto Rico (data from Puerto Rico were excluded from this analysis) [10]. This data atlas curates data from public sources to capture multiple domains of social drivers of health and health outcomes at several geographic units of analysis (including county level). Data included in the present study from the UCSF Health Atlas come from the following sources for the year 2020, which are delineated by variable in Supplementary Table S1: American Community Survey (ACS) 5-Year Estimates, Centers for Disease Control and Prevention PLACES, Housing and Urban Development Comprehensive Housing Affordability Strategy Data, and the Health Equity Action Network. The UCSF Health Atlas is available at healthatlas.ucsf.edu.

### 2.2. Study Variables

Our primary independent variable of interest was PRCDA county status per IHS 2020 definitions; a secondary variable of interest was Urban Indian Health Organization (UIHO) county status per IHS 2020 definitions. UIHO counties are those in which AIAN people in urban counties receive IHS care [11]. All analyses were conducted at the county level.

Dependent variables were classified into four groups according to domain: demographic and socioeconomic characteristics (age, sex, race, poverty, social vulnerability index, Gini index of income inequality, proportions receiving snap benefits, experiencing food insecurity, utility services threat, median income, unemployment, education, lack of broadband access, lack of computer access, lack of reliable transportation, and no automobile access); neighborhood and environment characteristics (housing insecurity, overcrowding, neighborhood deprivation index, severe rent/mortgage burden, population density, and proportion living in rural areas); healthcare access and utilization, and behaviors (uninsured, Medicaid enrollment, annual checkup [medical and dental], mammography, colorectal cancer screening, binge drinking, smoking, physical inactivity, social isolation, and social support); and health outcomes (disability, depression, poor mental health, arthritis, cancer, poor self-rated health, high blood pressure, coronary heart disease, diabetes, high cholesterol, obesity, stroke).

### 2.3. Statistical Analysis

We conducted descriptive ecological analyses comparing the mean (standard deviations) of county-level dependent variables between PRCDA and non-PRCDA counties. Differences between means were tested using *t*-tests; the equality of variances was examined, and for cases with unequal variance, Welch’s *t*-test was used. UIHO counties were not included in statistical comparisons because UIHO counties are considered a subset of PRCDA counties (n = 133). Effect sizes for pairwise comparisons were calculated using Cohen’s d statistic [12]. Unpooled standard deviations were used for calculating Cohen’s D statistic to account for heteroscedasticity. Cohen’s d statistics were interpreted as Small: |0.2–0.5|, Medium: |0.5–0.8|, and ≥|0.8|. Results were considered statistically significant at *p* < 0.05 with a Bonferroni corrected *p*-value, calculated using the formula: adjusted pi = min(pi × m, 1) [13]. Tables give both uncorrected and corrected *p*-values. All statistical analyses were conducted using SAS version 9.4 (SAS Institute Inc. Cary, NC, USA).

### 2.4. Ethics

This study presents publicly available county-level aggregate data; therefore, IRB review was not required.

## 3. Results

Table 1 gives average county-level demographic and socioeconomic characteristics across PRCDA, non-PRCDA, and UIHO counties with a statistical comparison between PRCDA and non-PRCDA counties. Compared to non-PRCDA counties, PRCDA counties had higher average proportions of the population identifying as AIAN (9.6 vs. 1.7%), unemployed (3.1 vs. 2.7%), and with a bachelor’s degree (57.4 vs. 53.6%). In contrast, PRCDA counties had lower average proportions of the population who were aged 19–64 years (57.9 vs. 58.5%), female (49.2 vs. 49.6%), experiencing a utility services threat (8.2 vs. 8.7%), with less than a high school education (10.5 vs. 12.0%), and lacking computer access (8.9 vs. 9.8%). In contrast, there were no statistically significant differences in poverty, income inequality, the percentage receiving SNAP benefits, food insecurity, median income, lack of broadband access, and transportation access.

Table 1 also gives characteristics of UIHO counties, a subset of PRCDA counties that are designated urban. While not compared statistically, due to the overlap between PRCDA and UIHO counties, there were some notable differences. For example, the median income was notably higher in UIHO counties ($84,358) when compared to non-PRCDA counties ($62,949). Perhaps reflecting this difference in income, poverty was also lower in UIHO counties (11.2% vs. 14.3% in non-PRCDA counties). The proportion of individuals with a less than high school education was lower, and the proportion with a bachelor’s degree or higher was higher in UIHO counties compared to non-PRCDA counties. Finally, the lack of access to broadband and computers was lower in UIHO counties compared to non-PRCDA counties.

Table 2 gives average county-level neighborhood and environment characteristics across PRCDA, non-PRCDA, and UIHO counties, with a statistical comparison between PRCDA and non-PRCDA counties. We observed statistically significantly higher average overcrowding (1.0 vs. 0.6%) and severe rent/mortgage burden (7.4 vs. 6.9%) among PRCDA counties compared to non-PRDA counties. There were no statistically significant differences in neighborhood deprivation index, population density, or the proportion of people living in rural areas.

Table 2 also gives average county-level characteristics for UIHO counties. As expected, the population density was higher in UIHO counties (960.9 people per km^2^), compared to non-PRCDA (116.4) counties, and the proportion of people living in rural areas was lower in UIHO counties (18.9) compared to non-PRCDA (64.6) counties.

Table 3 gives average county-level healthcare access and utilization characteristics across PRCDA, non-PRCDA, and UIHO counties with a statistical comparison between PRCDA and non-PRCDA counties. Compared to non-PRCDA counties, PRCDA counties had a higher average percentage uninsured under 19 years old (6.9 vs. 6.1%), with a recent dental visit (60.3 vs. 57.7%), reporting binge drinking (16.9 vs. 16.6%), and reporting social isolation (33.5 vs. 32.7%). In contrast, PRCDA counties had lower average percentages of those reporting an annual checkup (75.1 vs. 77.0%), women aged 50–74 y reporting a recent mammography (73.4 vs. 74.2%), current smokers (16.6 vs. 17.4%), and reporting physical inactivity (26.4 vs. 28.1%). There were no statistically significant differences in the percentage uninsured, the percentage enrolled in Medicaid, the percentage reporting colorectal cancer screening, or those reporting a lack of social and emotional support.

Table 3 also gives average county-level characteristics for UIHO counties. While not compared statistically due to the overlap between PRCDA and UIHO counties, we did observe slight variations in the average proportion of individuals reporting healthcare access and/or behaviors. For example, compared to non-PRCDA counties, UIHO counties had a lower proportion uninsured (7.6 vs. 9.4%), with an annual checkup (19.3 vs. 77.0%), current smoking (13.3 vs. 16.6%), physical inactivity (22.4 vs. 28.1%), a higher proportion with dental visits in the last year (64.3 vs. 57.7%), and reporting binge drinking (18.4 vs. 16.6%).

Table 4 gives average county-level neighborhood and environment characteristics across PRCDA, non-PRCDA, and UIHO counties, with a statistical comparison between PRCDA and non-PRCDA counties. Compared to non-PRCDA counties, PRCDA counties had lower average percentages of residents with poor mental health (17.0 vs. 17.3%), arthritis (30.7 vs. 31.4%), poor self-rated health (20.1 vs. 21.2%), high blood pressure (36.4 vs. 37.9%), diabetes (12.9 vs. 13.7%), high cholesterol (36.0 vs. 37.9%), and obesity (36.6 vs. 37.9%). There were no statistically significant differences in the percentage reporting disability, depression, cancer, and coronary heart disease.

Table 4 also gives average county-level characteristics for UIHO counties. Compared to PRCDA counties, a lower proportion of individuals in UIHO counties reported having disability (12.4 vs. 16.3%), arthritis (25.7 vs. 31.4%), poor self-rated health (17.0 vs. 21.2%), high blood pressure (31.2 vs. 37.9%), diabetes (10.8 vs. 13.7%), high cholesterol (33.9% vs. 37.1%), and obesity (32.5 vs. 37.9%).

## 4. Discussion

In this descriptive, ecological study, we explored differences in county-level demographic, socioeconomic, healthcare access, and health outcomes data between PRCDA and non-PRCDA counties, using data compiled within the USCF Health Atlas [11]. Although not part of our statistical comparisons, we also describe the prevalence of these same characteristics among UIHO counties, in which AIAN people in urban counties receive IHS care [11]. The purpose of this analysis was to understand contextual differences between PRCDA and non-PRCDA counties, given that cancer and other health surveillance statistics almost uniformly exclude AIAN people who reside in non-PRCDA counties due to concerns regarding racial misclassification [7]. We observed statistically significant differences between several variables across demographic, neighborhood, economic, and health outcomes, although not all of these differences were of large enough magnitude to be considered clinically meaningful. Nevertheless, while these data suggest that individuals living in PRCDA counties may experience different sociodemographic, economic, and healthcare contexts than those living in non-PRCDA counties, caution is warranted in the interpretation of these findings, given that the data are ecological and may not speak directly to the experience of AIAN people. It is also important to note that we observed no differences in several variables that are important drivers of health and healthcare access, including poverty, social vulnerability, food insecurity, and lack of social and emotional support. This study adds to the growing body of literature that serves as a call to action for improving AIAN racial misclassification in the cancer registry system and elsewhere, to provide improved cancer data for AIAN people. PRCDA resident status has served as a proxy for data quality (i.e., racial misclassification), restricting national statistics to those areas with a lower likelihood of racial misclassification; however, the results presented herein suggest that such a restriction may also create a selection bias in those included, based on structural and/or geographical differences between those included and excluded from estimates. Future work should consider understanding the effects of such selection bias on cancer burden statistics (e.g., incidence, mortality, and survival); for example, conducting quantitative bias analysis or similar.

Several methods have been used to address racial misclassification among AIAN people in population-based cancer registries in support of presenting data for all AIAN people, regardless of residence (or not) in PRCDA areas [14]. As noted in a recent systematic review on this topic [15], linkage with the IHS role is the most cited method for addressing AIAN racial misclassification in papers examining AIAN cancer statistics [2,15,16]. Since the method was introduced and validated in 2008 [17], IHS linkage has been a routine part of state-level cancer surveillance operations. Yet, linkage to the IHS roll is only effective at identifying those with a record of IHS enrollment; thus, the effectiveness of this method is lower in non-PRCDA counties, where individuals may be less likely to have received care from the IHS [6]. Further, IHS linkage is likely to become less effective over time as such individuals age into cancer diagnoses. Alternative methods include linkage to Tribal registries and/or Tribal rolls [18,19]; however, such approaches have only yet been explored at the local level, where Indigenous data sovereignty can be upheld for each Tribal Nation involved, rather than across the cancer surveillance system as a whole, which includes 574 federally recognized Tribal Nations and non-federally recognized nations nationwide. Findings from this and other research indicate the need for a reinvigorated effort to comprehensively address this issue.

In our findings, we observed that while some county-level characteristics were similar between PRCDA and non-PRCDA counties (e.g., prevalence of transportation barriers, housing insecurity), indicating similarities between PRCDA and non-PRCDA counties, other characteristics showed much larger differences (e.g., percentage of the population identifying as AIAN). Given that PRCDA counties are defined based on adjacency to Native reservations, Tribal lands, and IHS service areas [1,20], it might be expected that the proportion of the population identifying as AIAN would be higher in PRCDA versus non-PRCDA counties, as we report. Other social and structural drivers of health also differed. Some differences were statistically significant, while small (<1.0%), including PRCDA counties experiencing higher average overcrowding, severe rent/mortgage burden, utility services threat, and unemployment; others were much larger, including the proportion with a high school or college degree, and the social vulnerability index. These findings indicate ecological, county-level differences in the sociodemographic and economic context experienced by AIAN peoples, which may be important to cancer outcomes, should these findings directly reflect the AIAN experience, given the known impact of upstream social and structural conditions on downstream health outcomes [21].

County-level data on health-related factors are harder to interpret, since the data represent county averages that are not race-specific. For example, the prevalence of many self-reported health outcomes (poor self-rated health, high blood pressure, diabetes, high cholesterol, obesity) was lower in PRCDA counties, compared to non-PRCDA counties. Further, access to primary care and cancer screening was also lower in PRCDA compared to non-PRCDA counties. Given concerns regarding the underfunding of the IHS and its impact on healthcare access and utilization [22], it may not be surprising to find that these patterns also hold among AIAN peoples; however, county-level AIAN-specific data are not available for many of the data items presented herein to confirm. Increased race-specific data on social drivers of health and downstream factors (e.g., behaviors and health outcomes) are needed, but complicated by small population counts and the ability of existing federal surveys to recruit representative samples.

It should be noted that some factors presented herein that may be assumed to have a negative impact on health from a Westernized perspective may be interpreted differently using an Indigenous lens. For example, “overcrowding” is often associated with hazardous dwelling conditions [23] and/or infectious disease risk [24], as well as poverty [25], a major social driver of health [26]. Yet, “overcrowding” may also include multigenerational residence [27], which has shown mixed associations with mental and physical wellbeing [28]. Further, poverty itself is measured using a Westernized approach, and may not reflect non-cash wealth associated with Indigenous communities [29]. Collaboration with Tribal Nations to pursue decolonized approaches to epidemiology and health surveillance [30,31,32], including survey questions that more accurately reflect Indigenous wealth and resilience, may be needed to more accurately capture the social, demographic, and economic context of AIAN peoples than presented herein. Additionally, one may benefit from considering broader interpretations of these variables than have been employed heretofore.

Finally, in addition to indicating how the restriction of national statistics to PRCDA-resident AIAN peoples may be biasing national rates, these data also hint at why one might expect to see differences in cancer rates between AIAN people resident in PRCDA counties and those in non-PRCDA counties. Several of the factors presented herein are related to cancer risk; for example, alcohol use has been linked with increased risk of breast, liver, colorectal, oral, and stomach cancers [33,34], while tobacco smoking has been causally linked to thirteen different types of cancer, causing about 20% of all cancers in the U.S. [35]. In contrast, physical activity is strongly associated with reduced risk of at least seven different cancers, including breast, colon, endometrial, gastric, and renal cancers [36]. Unfortunately, due to differential racial misclassification resulting in higher suppression of cancer rates in non-PRCDA counties (i.e., higher racial misclassification in non-PRCDA counties resulting in lower case counts, and greater artificial suppression of cancer rates) [7], it will be difficult to determine whether observed differences in rates between AIAN people living in PRCDA and non-PRCDA counties are artificial—due to racial misclassification—or real, a result of the sociodemographic and economic differences presented herein.

The major limitations of this work are that it provides only county-level averages for each factor studied and does not speak specifically to the AIAN experience. PRCDA counties are designated by the IHS to identify those counties within which residents are eligible for IHS Purchased/Referred Care. Both Native and non-Native individuals reside in PRCDA counties across the U.S.; therefore, the data presented herein include data from all residents of PRCDA or non-PRCDA counties, regardless of race/ethnicity. AIAN-specific patterns within the county may diverge from the aggregate measures presented herein; furthermore, within-county variation in each of these measures is likely. Unfortunately, AIAN-specific data are not available for most of the measures herein, as these data are pulled from national data sources including the American Community Survey, the decennial Census, and the Behavioral Risk Factor Surveillance Survey (through CDC Places, which models county-level data based on survey responses). Given the small size of the AIAN population, it is likely not possible to provide county-level data for many of these measures specifically for AIAN people. However, as others have noted [37], the size of a population should not determine the importance of ensuring comprehensive and accurate surveillance data to support population health and well-being, particularly for underserved and minoritized populations such as AIAN people. Indeed, the data presented herein may support the need for additional research on this topic, perhaps focused on local data collection by and for AIAN people; such research would be in line with calls for decolonialized approaches to research and Tribal sovereignty of data (collection) [30,31,38]. Another limitation includes the restriction of our analyses to variables that were publicly available through the UCSF Health Atlas. While this data source is comprehensive on many levels, it is also limited by the data sources it pulls from. For example, we discuss above the potentially surprising finding that PRCDA and non-PRCDA counties were comparable in their proportion of rural residents; given that there are multiple definitions of rurality [39], one might see differences if using a different definition than the one presented herein (which defined urban as encompassing at least 2000 housing units or having a population of at least 5000) [10], or by using a different spatial level than county. Further, due to the descriptive ecological design, we were not able to account for potential correlation among variables or adjust for confounding. For example, herein, we did not examine differences by region, which we know is important to both AIAN cultures and cancer burden [1,2], and which may explain some of the differences observed herein. Future work should extend that presented herein by exploring differences in the socioecological context by region. Finally, PRCDA-designated counties do vary over time; thus, the data provided herein may not reflect county-level context relevant to historical cancer statistics.

## 5. Conclusions

The data presented herein indicate variability in county-level sociodemographic characteristics, area-based social measures, and prevalence of health outcomes between PRCDA and non-PRCDA counties. While these data do not speak specifically to the AIAN experience, they provide critical contextual information that informs our understanding of whether the exclusion of non-PRCDA AIAN people from cancer statistics may bias estimates. The collection of data specific to the AIAN population will be needed to fully understand the potential for and impact of this bias. Further, future work could include a quantitative bias analysis or similar to understand the effect of restricting national statistics to AIAN people living only in PRCDA counties. Efforts are needed (and ongoing) to understand how to better address racial misclassification among AIAN people within the cancer surveillance system, including improving and/or extending data linkages, which will allow for more accurate cancer statistics for those living in both PRCDA and non-PRCDA counties. In the meantime, researchers may consider presenting data stratified by PRCDA status (recognizing that particularly non-PRCDA rates are likely to be underestimates), and/or acknowledging differences in social context and cancer burden between AIAN peoples resident in PRCDA and non-PRCDA areas.

## Figures and Tables

**Table 1:** Demographic and socioeconomic characteristics of PRCDA, UIHO, and non-PRCDA counties, comparing PRCDA and non-PRCDA counties. Derived from the UCSF Neighborhood Health Atlas ^a,b^.

Variable	Non PRCDA N = 2472		PRCDA N = 680		UIHO N = 133		Cohens d Effect Size Comparing PRCDA to Non-PRCDA	Bonferroni Adjusted *p*-Value Comparing PRCDA to Non PRCDA
	N	Mean (SD)	N	Mean (SD)	N	Mean (SD)		
Demographic								
Age								
Age < 18 y, (%)	2472	21.8 (3.3)	672	22.1 (4.3)	133	22.5 (3.4)	−0.067	1.000
Age 19–64 y, (%)	2472	58.5 (4.0)	672	57.9 (4.2)	133	61.3 (3.3)	0.146	**0.041**
Age 65+ y, (%)	2472	19.6 (4.6)	672	20.0 (5.6)	133	16.2 (4.0)	−0.065	1.000
Female, (%)	2472	49.6 (2.5)	672	49.2 (2.4)	133	50.0 (1.3)	0.160	**0.012**
American Indian and Alaska Native (%)	2472	1.7 (2.6)	672	9.6 (16.0)	133	3.9 (6.7)	−0.692 ^c^	**<0.0001**
Socioeconomic								
Poverty								
Poverty among all individuals (%)	2472	14.4 (6.0)	672	14.3 (6.0)	133	11.2 (4.5)	0.010	1.000
Poverty among individuals under 18 y (%)	2471	19.0 (9.9)	672	18.6 (9.2)	133	13.9 (6.5)	0.039	1.000
Poverty among individuals over 65 y (%)	2472	10.8 (4.7)	672	10.3 (4.5)	133	9.2 (3.5)	0.108	0.654
Social vulnerability index	2463	0.49 (0.29)	680	0.53 (0.28)	133	0.48 (0.28)	−0.150	**0.029**
Gini index of income inequality	2472	0.45 (0.04)	672	0.44 (0.03)	133	0.45 (0.04)	0.069	1.000
SNAP benefits (%)	2472	12.4 (6.4)	672	12.3 (6.2)	133	10.0 (5.1)	0.012	1.000
Food insecurity (%)	1888	15.7 (5.9)	529	15.0 (5.8)	108	13.3 (4.3)	0.117	0.883
Utility services threat (%)	1888	8.7 (2.8)	529	8.2 (2.9)	108	7.5 (2.2)	0.178	**0.016**
Median income ($)	2471	62,949 (17,241)	672	64,648 (15,111)	133	84,358 (21,553)	−0.105	0.631
Unemployment (%)	2472	2.7 (1.2)	672	3.1 (1.7)	133	3.2 (1.1)	−0.230 ^d^	**<0.0001**
Less than high school (%)	2472	12.0 (5.9)	672	10.5 (4.9)	133	9.4 (4.8)	0.276 ^d^	**<0.0001**
Bachelor’s or higher (%)	2472	53.6 (10.9)	672	57.4 (9.5)	133	66.6 (8.3)	−0.368 ^d^	**<0.0001**
Digital access								
Lack of broadband access (%)	2472	17.7 (7.2)	672	17.1 (7.3)	133	10.3 (3.8)	0.077	1.000
Lack of computer access (%)	2472	9.8 (4.7)	672	8.9 (4.5)	133	5.1 (2.4)	0.196 ^d^	**0.001**
Lack of reliable transportation (%)	1888	9.1 (2.7)	529	9.2 (3.0)	108	8.3 (2.1)	−0.032	1.000
No automobile access (%)	2472	5.9 (3.7)	672	6.4 (6.3)	133	8.0 (10.2)	−0.098	0.409

^a^ Column values are mean (standard deviation) of county-level values. Rows indicate how that variable is measured, e.g., as a percentage, or an index. ^b^ Values in bold are considered statistically significant at the Bonferroni-adjusted *p*-value, with formula: adjusted pi = min(pi × m, 1). ^c^ Cohen’s d value 0.5–0.8, indicating a medium effect size. ^d^ Cohens d value 0.2–0.5, indicating a small effect size.

**Table 2:** Neighborhood and environment characteristics of PRCDA, UIHO, and non-PRCDA counties, comparing PRCDA and non-PRCDA counties. Derived from the UCSF Neighborhood Health Atlas ^a,b^.

Variable	Non PRCDA N = 2472		PRCDA N = 680		UIHO N = 133		Cohen’s d Effect Size	Bonferroni Adjusted *p*-Value Comparing PRCDA to Non PRCDA
	N	Mean (SD)	N	Mean (SD)	N	Mean (SD)		
Housing insecurity (%)	1888	12.8 (4.1)	529	12.5 (4.0)	108	12.0 (3.3)	0.068	1.000
Overcrowding (%)	2472	0.6 (0.8)	672	1.0 (1.7)	133	1.2 (1.1)	−0.282 ^c^	**<0.0001**
Neighborhood deprivation index	2467	−0.08 (0.90)	672	−0.12 (0.90)	133	−0.21 (0.84)	0.044	1.000
Severe mortgage/rent burden (%)	2471	6.9 (2.6)	672	7.4 (2.7)	133	8.4 (3.1)	−0.178	**0.002**
Population density (people per km^2^)	2463	116.4 (800.8)	672	71.6 (274.9)	133	960.9 (3179)	0.075	1.000
People living in rural areas (%)	2463	64.6 (33.4)	672	62.6 (34.3)	133	18.9 (24.6)	0.058	1.000

^a^ Column values are mean (standard deviation) of county-level values. Rows indicate how that variable is measured, e.g., as a percentage or an index. ^b^ Values in bold are considered statistically significant at the Bonferroni-adjusted *p*-value, with formula:adjusted pi = min(pi × m, 1). ^c^ Cohen’s d value 0.2–0.5, indicating small effect size.

**Table 3:** Healthcare access and utilization, and health-related behaviors of PRCDA, UIHO, and non-PRCDA counties, comparing PRCDA and non-PRCDA counties. Derived from the UCSF Neighborhood Health Atlas ^a,b^.

Variable	Non PRCDA N = 2472		PRCDA N = 680		UIHO N = 133		Cohen’s d Effect Size	Bonferroni Adjusted *p*-Value Comparing PRCDA to Non PRCDA
	N	Mean (SD)	N	Mean (SD)	N	Mean (SD)		
Healthcare access and utilization								
Uninsured (%)	2472	9.4 (5.0)	672	9.9 (5.4)	133	7.6 (3.6)	−0.088	1.000
Uninsured among under 19 y (%)	2471	6.1 (5.4)	672	6.9 (5.3)	133	4.7 (3.0)	−0.147	**0.037**
Enrolled in Medicaid (%)	2472	21.5 (8.1)	672	22.3 (8.4)	133	19.3 (8.8)	−0.105	0.833
Annual Checkup (%)	2472	77.0 (3.2)	672	75.1 (4.3)	133	73.6 (3.9)	0.498 ^c^	**<0.0001**
Dental visit in the past year among adults (%)	2472	57.7 (11.1)	672	60.3 (9.4)	133	64.3 (5.7)	−0.253 ^d^	**<0.0001**
Mammography among women aged 50–74 y (%)	2472	74.2 (3.9)	672	73.4 (5.1)	133	75.8 (4.3)	0.177	**0.001**
Colorectal cancer screening among adults aged 50–75 y (%)	2472	63.8 (4.4)	672	63.8 (5.7)	133	63.6 (4.5)	0.001	1.000
Health-related behaviors								
Binge drinking (%)	2472	16.6 (2.5)	672	16.9 (2.6)	133	18.4 (2.4)	−0.147	**0.037**
Smoking (%)	2472	17.4 (3.7)	672	16.6 (4.1)	133	13.3 (3.1)	0.193	**<0.0001**
Physical inactivity (%)	2472	28.1 (5.3)	672	26.4 (5.3)	133	22.4 (4.5)	0.324 ^d^	**<0.0001**
Social isolation (%)	2472	32.7 (2.5)	672	33.5 (3.3)	133	33.8 (2.7)	0.324 ^d^	**<0.0001**
Lack of social and emotional support (%)	1888	25.0 (4.0)	529	24.9 (3.8)	108	25.3 (3.8)	0.027	1.000

^a^ Column values are mean (standard deviation) of county-level values. Rows indicate how that variable is measured, e.g., as a percentage, or an index. ^b^ Values in bold are considered statistically significant at the Bonferroni-adjusted *p*-value, with formula:adjusted pi = min(pi × m, 1). ^c^ Cohen’s d value 0.5–0.8, indicating medium effect size. ^d^ Cohens d value 0.2–0.5, indicating small effect size.

**Table 4:** Health outcomes in PRCDA, UIHO, and non-PRCDA counties, comparing PRCDA and non-PRCDA counties. Derived from the UCSF Neighborhood Health Atlas ^a,b^.

Variable	Non PRCDA N = 2472		PRCDA N = 680		UIHO N = 133		Cohens d Effect Size	Bonferroni Adjusted *p*-Value Comparing PRCDA to Non PRCDA
	N	Mean (SD)	N	Mean (SD)	N	Mean (SD)		
Disability (%)	2472	16.3 (4.7)	672	15.8 (4.0)	133	12.4 (2.8)	0.103	0.685
Depression (%)	2472	23.2 (3.4)	672	23.2 (3.2)	133	22.2 (3.1)	−0.014	1.000
Poor mental health (%)	2472	17.3 (2.3)	672	17.0 (2.3)	133	16.2 (1.9)	0.144	**0.047**
Arthritis (%)	2472	31.4 (4.8)	672	30.7 (4.6)	133	25.7 (4.0)	0.156	**0.018**
Cancer (%)	2472	9.0 (1.5)	672	9.1 (1.7)	133	7.9 (1.5)	−0.017	1.000
Poor self-rated health (%)	2472	21.2 (5.1)	672	20.1 (4.4)	133	17.0 (3.7)	0.226 ^c^	**<0.0001**
High blood pressure (%)	2411	37.9 (5.5)	666	36.4 (5.2)	133	31.2 (4.0)	0.282 ^c^	**<0.0001**
Coronary heart disease (%)	2472	8.4 (1.6)	672	8.3 (1.6)	133	6.5 (1.2)	0.076	1.000
Diabetes (%)	2472	13.7 (2.8)	672	12.9 (2.4)	133	10.8 (1.9)	0.301 ^c^	**<0.0001**
High cholesterol (%)	2411	37.1 (3.2)	666	36.0 (3.3)	133	33.9 (3.0)	0.328 ^c^	**<0.0001**
Obesity (%)	2472	37.9 (4.5)	672	36.6 (4.9)	133	32.5 (5.5)	0.268 ^c^	**<0.0001**
Stroke (%)	2472	4.3 (0.9)	672	4.3 (0.9)	133	3.3 (0.6)	−0.002	1.000

^a^ Column values are mean (standard deviation) of county-level values. Rows indicate how that variable is measured, e.g., as a percentage, or an index. ^b^ Values in bold are considered statistically significant at the Bonferroni-adjusted *p*-value, with formula:adjusted pi = min(pi × m, 1). ^c^ Cohen’s d value 0.2–0.5, indicating small effect size.

## Data Availability

Data are available from the University of California, San Francisco Health Atlas at https://healthatlas.ucsf.edu (accessed on 24 March 2026).

## Supplementary Materials

The following supporting information can be downloaded at: https://www.mdpi.com/article/10.3390/ijerph23050622/s1, Supplementary Table S1. Variables included in the present analysis (2020 data downloaded from the UCSF Health Atlas), with descriptions. Adapted from the UCSF Health Atlas Data Dictionary.

## Abbreviations

AIAN: American Indian and Alaska Native; IHS: Indian Health Service; PRCDA: Purchased/Referred Care Delivery Area; UIHO: Urban Indian Health Organization; UCSF: University of California, San Francisco; IRB: Institutional Review Board; DC: District of Columbia; ACS: American Community Survey; SAS: Statistical Analysis System; US: United States.
