# Supplementary material for: Area-Level Sociodemographic Differences Between Indian Health Service Purchased/Referred and Non-Purchased/Referred Care Delivery Areas
Source: Int J Environ Res Public Health. 2026 May 8;23(5):622. doi: 10.3390/ijerph23050622 (PMC13205842; doi:10.3390/ijerph23050622)
Supplement: Supplementary file 1 [file ijerph-23-00622-s001.zip › ijerph-4250503-supplementary.pdf]

**Supplementary Table S1.** Variables included in the present analysis (2020 data downloaded from the UCSF Health Atlas), with descriptions. Adapted from the UCSF Health Atlas Data Dictionary.[a]

| Variable Name | Variable description | units | source |
|---|---|---|---|
| *Demographic* | | | |
| Under 18 years old | Percent of population under 18 years old | percent | ACS |
| 18-64 years old | Percent of population 18-64 years old | percent | ACS |
| Over 65 years old | Percent of population 65 years and over | percent | ACS |
| Female | Percent of the population who are female | percent | ACS |
| Male | Percent of the population who are male | percent | ACS |
| American Indian or Alaska Native | Percent of the population who are American Indian or Alaska Native alone or in combination with one or more other races | percent | ACS |
| *Socioeconomic* | | | |
| Poverty among all individuals | Percent of the population with income below poverty level in the past 12 months | percent | ACS |
| Poverty among individuals under 18 | Percent of the population under 18 living in households with income below poverty level in the past 12 months | percent | ACS |
| Poverty among individuals over 65 | Percent of the population over 65 with income below poverty level in the past 12 months | percent | ACS |
| Median income | Median gross household income (dollars) | estimate | ACS |
| Food insecurity | Food insecurity in the past 12 months among adults 18 and older | percent | PLACES |
| SNAP benefits | Percent of the population with Food Stamp/SNAP benefits in the past 12 months | percent | ACS |
| Utility services threat | Threat of utilities shut off in past 12 months among adults 18 and older | percent | PLACES |
| Less than high school | Percent of the population with a less than high school education | percent | ACS |

| | | | |
|---|---|---|---|
| Some college or more | Percent of the population with some college or more | percent | ACS |
| Gini Index of Income Inequality | Gini index of income inequality, where 0 indicates perfect equality and 1 indicates perfect inequality | index | ACS |
| Neighborhood Deprivation Index | Neighborhood deprivation index, where higher values indicate greater neighborhood deprivation | index | Health Equity Action Network |
| Neighborhood Deprivation Index Quintiles | Neighborhood deprivation index quintiles where 1 is lowest and 5 is highest deprivation | quintile | Health Equity Action Network |
| Social Vulnerability Index | Percentile ranking (0 to 100) with higher values indicating greater vulnerability) | index | CDC |
| Households without broadband internet | Percent of households without a broadband internet | percent | ACS |
| Households without a computer | Percent of households without a computer | percent | ACS |
| Unemployed | Percent of population 16 and over in civilian labor force that is unemployed | percent | ACS |
| Extremely low-income households | Percent of households making less than 30% of the HUD Area Median Family Income | percent | HUD |
| *Health Outcomes* | | | |
| Disability | Percent of total population with a disability | percent | ACS |
| Arthritis | Arthritis among adults 18 and older | percent | PLACES |
| All Cancers excluding skin | Cancer (excluding skin cancer) among adults 18 and older | percent | PLACES |
| Poor self-rated health | Fair or poor self-rated health status among adults 18 and older | percent | PLACES |
| High blood pressure | High blood pressure among adults 18 and older | percent | PLACES |
| Asthma | Current asthma among adults 18 and older | percent | PLACES |

| | | | |
|---|---|---|---|
| Coronary heart disease | Coronary heart disease among adults 18 and older | percent | PLACES |
| Chronic obstructive pulmonary disease | Chronic obstructive pulmonary disease among adults 18 and older | percent | PLACES |
| Diabetes | Diagnosed diabetes among adults 18 and older | percent | PLACES |
| High cholesterol | High cholesterol among adults 18 and older | percent | PLACES |
| Obesity | Obesity among adults 18 and older | percent | PLACES |
| Poor physical health | Physical health not good for over 14 days among adults 18 and older | percent | PLACES |
| Stroke | Stroke among adults 18 and older | percent | PLACES |
| Depression | Depression among adults 18 and older | percent | PLACES |
| Poor mental health | Mental health not good for over 14 days among adults 18 and older | percent | PLACES |
| *Healthcare access and utilization* | | | |
| Binge drinking | Binge drinking among adults 18 and older | percent | PLACES |
| Current smoking | Current smoking among adults 18 and older | percent | PLACES |
| Physical inactivity | No leisure-time physical activity among adults 18 and older | percent | PLACES |
| Sleep less than 7 hours | Sleeping less than 7 hours among adults 18 and older | percent | PLACES |
| Uninsured | Percent of the population with no health insurance coverage | percent | ACS |
| Uninsured among under 19 | Percent of the population 0-18 years old with no health insurance coverage | percent | ACS |
| Enrolled in Medicaid | Percent of population enrolled in Medicaid health insurance coverage | percent | ACS |
| Annual checkup | Visits to doctor for routine checkup within the past year among adults 18 and older | percent | PLACES |

| | | | |
|---|---|---|---|
| Dental visit | Visits to dentist or dental clinic among adults 18 and older | percent | PLACES |
| Mammography | Mammography use among women aged 50‚Äì74 years | percent | PLACES |
| Colorectal cancer screening | Fecal occult blood test, sigmoidoscopy, or colonoscopy among adults aged 50-75 years | percent | PLACES |
| *Neighborhood* | | | |
| Limited English proficiency | Percent of the population that speaks English less than "very well" | percent | ACS |
| Speak language other than English at home | Percent of the population that speaks a language other than English at home | percent | ACS |
| Housing insecurity | Housing insecurity in the past 12 months among adults 18 and older | percent | PLACES |
| Vacant housing units | Percent of total housing units that are vacant | percent | ACS |
| Overcrowding | Percent of households with 1.51 or more occupants per room | percent | ACS |
| Severe mortgage burden among owner-occupied households | Percent of owner-occupied households that are paying more than 50% of their income to housing costs | percent | HUD |
| Severe rent burden among renter-occupied households | Percent of renter-occupied households that are paying more than 50% of their income to housing costs | percent | HUD |
| Severe rent burden among extremely low income households | Percent of extremely low income households that are paying more than 50% of their income to housing costs | percent | HUD |
| Lack of reliable transportation | Lack of reliable transportation in the past 12 months among adults 18 and older | percent | PLACES |
| No automobile access | Percent of households with no vehicle | percent | ACS |
| Social isolation | Feelings of social isolation among adults 18 and older | percent | PLACES |
| Lack of social and emotional support | Lack of social and emotional support among adults 18 and older | percent | PLACES |

| | | | |
|---|---|---|---|
| Population density | Number of people living within 1 square kilometer of land | estimate | U.S. Census |
| People living in rural areas | Percent of the population living in rural areas | percent | U.S. Census |

[a]Abbreviations: ACS: American Community Survey; HUD: Housing and Urban Development; PLACES: Centers for Disease Control and Prevention PLACES.
